# Defining young people’s mental health self-care: a systematic review and co-development approach

**DOI:** 10.1007/s00787-023-02320-7

**Published:** 2023-11-10

**Authors:** Alex Truscott, Daniel Hayes, Tom Bardsley, Disha Choksi, Julian Edbrooke-Childs

**Affiliations:** 1 Evidence Based Practice Unit, University College London and Anna Freud National Centre for Children and Families, 4-8 Rodney St, London, N1 9JH UK; 2 Research Department of Behavioural Science and Health, Institute of Epidemiology and Health Care, University College London, 1-19 Torrington Place, London, WC1E 7HB UK; 3 Anna Freud National Centre for Children and Families, 4-8 Rodney St, London, N1 9JH UK

**Keywords:** Patient and public involvement, Systematic review, Youth, Self-care, Mental health, Wellbeing

## Abstract

Self-care is among the emerging types of mental health support which operate outside traditional services, although the meaning and practice of self-care for young people with mental health difficulties are currently unclear. This systematic review was pre-registered with PROSPERO (CRD42021282510) and investigated conceptualizations of self-care in academic publications which investigated or discussed self-care for young people’s mental health or wellbeing. A Patient and Public Involvement (PPI) workshop facilitated young people with experience of mental health difficulties to respond to the identified concepts and co-develop a definition of self-care. Searches in PsycINFO, MEDLINE, Embase, CINAHL Plus, Scopus, Cochrane Library of Systematic Reviews, and gray literature sources resulted in 90 included publications. Content analysis indicated little conceptual consistency, with health and wellness promotion most commonly used to define self-care. The PPI workshop co-developed a definition of mental health self-care, which attendees felt should emphasize an individual process of self-awareness, self-compassion, and specific strategies to work toward emotional balance. This study highlights the gap between current academic understandings of young people’s mental health self-care and young people’s experience. The presented definition will enable future research to begin from an understanding of self-care which is relevant to young people with experience of mental health difficulties.

**Supplementary Information:**

The online version contains supplementary material available at 10.1007/s00787-023-02320-7.

## Introduction

Recent evidence concerning young people’s mental health has consistently indicated high levels of need. For example, the last survey of young people’s mental health in England found that 12.8% of 5–19 year olds were experiencing at least one diagnosable mental health condition, with 5.8% experiencing an emotional disorder [1]. Additionally, the 2022 follow-up of the Mental Health of Children and Young People survey series in England reported findings that among the 7–16-year-old age group, 18% showed a probable mental disorder, with a slightly higher proportion reported for those 17–24 years (22%) [2].

Despite these rising prevalence rates, many young people with mental health difficulties do not receive support from mental health professionals [1]. Many reasons can result in a young person being largely self-reliant regarding their mental health, for example the impact of stigma, lack of awareness of symptoms or support, the young person’s preference or the availability of services [3, 4]. Considering the availability of statutory mental health support, the National Health Service in England is only able to provide support to approximately one in three young people with a diagnosable mental health condition [5]. Although other routes to professional support do exist, this represents a substantial gap between service availability and potential service need for young people with mental health difficulties.

A growing body of evidence has instead focused on non-professional and community-based approaches to mental health support [6]. These often focus on informal use of more easily accessible resources and have found benefits to mental health from activities such as spending time in nature [7, 8], creative arts [9] and exercise [10]. While ideally multiple forms of both professional and alternative mental health support would be accessible dynamically over time depending on the young person’s preferences and level of need, the relative lack of availability of professional mental health support may result in some young people increasingly relying on alternative forms of support.

These alternative approaches are often discussed separately, but tend to share common elements of being widely available and accessible based on individual choice. There are, of course, constraints on these choices with the availability of activities depending on factors such as a young person’s local area, potential financial barriers and knowledge of community support options. However, exploration of these approaches might be particularly relevant for young people experiencing a life stage of increased independence with both the opportunities and challenges this presents. Indeed, research with young people highlights the importance of agency and choice in determining how they manage their mental health [11], while a review of self-care in mental health services similarly identified choice, control, and engagement as critical in determining the types of self-care support an individual might need [12].

In this context, self-care may be a helpful organizing framework with the potential to support young people to bring together their available resources and current mental health and wellbeing needs, to develop an understanding of what works to support their mental health in everyday life. A greater understanding of how to support young people’s self-care would not suggest a lack of need for professional care, but would empower young people to use the resources in their everyday lives, regardless of any other support they were receiving. By seeking a greater understanding of young people’s mental health self-care, their expertise and the actions which they already take in relation to their mental health can be recognized and supported, alongside how different forms of support, such as self-care, school-based support, community support, and professional care, can all work together.

Academic definitions of self-care often refer to care for long-term physical health conditions, with one of the first definitions of self-care originating from nursing theory and defining the term as “the practice of activities that individuals initiate and perform on their own behalf in maintaining life, health, and wellbeing” [13]. From these roots in physical health, self-care for mental health has gained only gradual and partial recognition from many public health bodies. Considering the current definition used by the World Health Organization, it has a broader focus than physical health, but still without clearly including mental health: “Self-care is the ability of individuals, families and communities to promote health, prevent disease, and maintain health and to cope with illness and disability with or without the support of a health-care provider” [14].

Definitions of self-care specifically for mental health have been developed [e.g., 15]; however, reviews of self-care definitions across physical and mental health have struggled to identify a consistent conceptual basis for the term [16–19]. Alongside this lack of consensus in the academic literature, very little research has investigated whether these varying academic conceptualizations of self-care are consistent with young people’s understanding of self-care for mental health. While some studies have asked young people about the specific activities they use to look after their mental health [20, 21], few have sought young people’s views on the meaning of the term. Those that have include two studies with undergraduate students [22, 23] and one study of spiritual self-care with adolescents [24], which all focus on promoting wellbeing rather than managing mental health difficulties.

Further development of this evidence base will need an understanding of how the term has been used to date, to gain clarity on the concepts under study when authors report investigating ‘self-care’. Additionally, to ensure that future self-care research is relevant to young people with mental health difficulties, these academic conceptualizations will need to be informed by the lived experiences of young people who use mental health self-care. While multiple understandings of the term ‘self-care’ may exist, gaining greater clarity of current conceptualizations will help to identify differing uses of the term and support the field to progress by exploring the varying meanings of self-care in use across young people’s mental health and wellbeing literature. This will provide a basis for future self-care research to consider and identify their own conceptualization of self-care, enabling a clearer understanding of self-care research in this field.

Overall, the current study aimed to evaluate and extend academic conceptualizations of young people’s mental health self-care. Therefore, the study first sought to understand how self-care has been conceptualized in the young people’s mental health and wellbeing literature through how the term has been both defined and measured. The research questions guiding the systematic review were: (1) How has self-care been defined in the young people’s mental health and wellbeing literature? and (2) How has self-care been measured in the young people’s mental health and wellbeing literature? Second, we sought to use a Patient and Public Involvement approach to facilitate young people to respond to these conceptualizations and co-develop a definition that captured their experiences of mental health self-care.

## Methods

This systematic review was registered with PROSPERO (CRD42021282510) and the reporting is in accordance with the PRISMA 2020 Checklist [25]. The reporting of the Patient and Public Involvement (PPI) workshop is in accordance with the GRIPP2 short form checklist [26].

### Eligibility criteria

This systematic review aimed to explore conceptualizations of self-care in academic publications related to young people’s mental health and wellbeing. Therefore, publications were included if they met the following criteria: (1) self-care was for young people 11–25 years, (2) publication aimed to investigate or discuss self-care, (3) self-care was for mental health or wellbeing, (4) published 2000–present, (5) full text available in English, (6) published in an academic journal or gray literature, and (7) full-length papers or reports.

A broad definition of ‘young person’ was used to capture the age from which many young people start to experience independence at the beginning of secondary school to the upper age limit used by a number of young people’s services, including the NHS [27]. A wide range of ways of investigating self-care were also considered: (a) being a main point of discussion in non-empirical papers, (b) being investigated in empirical papers, (c) being part of an intervention or broader concept, or (d) as a more specific form of self-care such as professional self-care or self-care agency. This review did not start with a working definition of self-care to remain open to how authors from across the field conceptualized the term. Self-care was of interest wherever it was discussed or investigated in relation to young people’s mental health or wellbeing. Additionally, a broad perspective of mental health and wellbeing was used in order to capture an overall sense of how self-care was conceptualized in the field. Mental health included a focus anywhere on the spectrum from mentally healthy to unwell, while wellbeing was understood in accordance with the ‘thriving’ concept proposed by Ross, et al. [28]. These understandings did not include neurodevelopmental divergence, unless the self-care aimed to support general wellbeing or a co-occurring mental health difficulty.

Specialist organizations were included in the gray literature search to maintain a focus on academic rather than public conceptualizations of self-care. These organizations were selected to represent a range of perspectives, including public health bodies, general mental health organizations, and organizations specific to young people’s mental health. Reports identified through the specialist organization websites were evaluated against the same inclusion criteria as publications identified through academic databases.

### Search strategy

The overall search strategy was developed from scoping exercises and adapted following consultation with a specialist subject librarian. Searches were run across Medline, Embase, PsycINFO, CINAHL Plus, Scopus and the Cochrane Library of Systematic Reviews from 9th to 11th October 2021. An example search string is available as Online Resource 1.

Gray literature was searched through the BASE database of gray literature and the websites of NHS England, NICE, Gov.uk, Mental Health Foundation, Kings Fund, Young Minds, World Health Organization and Centre for Mental Health. These resources were last searched on 9th October 2021. Due to limited search complexity capabilities, most organization websites were searched for ‘self-care’ and ‘self care’. The reference lists of included publications were also hand-searched to identify any further relevant literature.

After removing duplicate publications, the searches of academic databases resulted in 9325 records, while the gray literature search resulted in 1748 records.

### Screening and selection

The publications identified through the search were first screened against the eligibility criteria based on their title and abstract. Where publications met the eligibility criteria or the title and abstract did not contain enough information, they were included to be assessed at the full text stage. Two reviewers screened publications against the eligibility criteria in this way, the first reviewer (A.T.) screened 100% of the publications and the second reviewer (T.B.) independently double screened 10% at the title and abstract stage. The conceptual confusion which prompted this review also resulted in challenges during the initial screening process, with an agreement rate of 90%. After discussion of disagreements, 1533 records from the academic database search and 111 records from the gray literature search were taken forward to the full text assessment stage.

Full texts were missing for 17 of these records, with publications indicated as ‘not retrieved’ when an initial and follow-up email to two study authors (where possible), a request to the British Library and a ResearchGate request were all unsuccessful in obtaining the full text.

A total of 1627 records were, therefore, assessed against the eligibility criteria based on their full text. The double screening rate was increased at the full text stage to ensure that final inclusion and exclusion decisions were consistent, with the second reviewer independently double screening 20% of publications. At this stage, the two reviewers demonstrated a substantial agreement rate of 98.2%.

### Analytic strategy

The data extraction table included the publication type, country, publication year, participant descriptions, any age information, study aim, role of self-care (if unclear from the aims), references for any definitions of self-care cited by each publication, the exact wording of any self-care definition given, and details of any self-care measurement. Data extraction was double checked by the second reviewer for 10% of included studies.

Data synthesis and analysis focused on publications which had either defined or measured self-care. References to other self-care definitions were also recorded to determine how frequently each was cited in the review sample. Content analysis was performed on NVivo to inductively analyze concepts underpinning the definitions and measurements. Publications which measured self-care were organized by type of measurement and separate coding sheets were drawn up for each, in addition to a coding sheet for definitions. Measurement types included interview questions, instructions for participant activities, systematic review search terms, deductive coding frameworks, validated measures, measures without published validity information (non-validated measures), and questionnaires developed specifically for the reported study (project-specific questionnaires).

Definitions and measurement of self-care through interview questions or written activity instructions were coded according to both the overall concepts and any specific activities mentioned. For publications which measured self-care through questionnaires or measures, the coding sheet was derived from the overall concept measured as stated in the description of the questionnaire or measure. Once definitions and measures of self-care had been coded, frequencies of each code were calculated and overlap between the coding frameworks was considered to understand the overall conceptualization of self-care and the most common concepts used across both of these domains.

Quality assessment methods were considered to be of limited value for this review. As data extraction focused on the introduction and methods sections of included publications, standard quality assessment tools which aim to evaluate trustworthiness of study findings lacked relevance to the study aims. Instead, the review itself provides a perspective on quality by investigating the clarity of conceptualization of self-care through presence or absence of definition and measurement, alongside the consistency of each conceptualization within the wider literature.

### Patient and Public Involvement workshop

The Patient and Public Involvement workshop aimed to evaluate and extend the conceptualizations of self-care explored in the systematic review. To facilitate young people to respond to the systematic review findings, an online workshop was run with five young people. The workshop was run with three co-facilitators, the first reviewer, a young people’s involvement specialist, and a young person. The workshop was attended by young people with experience of mental health difficulties who were engaged with a program run by a mental health charity to support young people’s input to services and research. The program is guided by the Lundy Model of Participation [29].

The session involved both open discussion of the meaning of mental health self-care and an activity whereby the young people evaluated the concepts identified by the systematic review. The concepts were presented online in a random order as tiles on a single page, which the young people were asked to rank on a five-point scale from ‘Completely irrelevant’ to ‘Completely relevant’ when considering their own understanding of mental health self-care.

Co-production of a definition of mental health self-care began by discussing the concepts which the young people had indicated as either ‘Completely relevant’ or ‘Slightly relevant’. These concepts were used to stimulate discussion rather than act as limits to the young people’s thinking and the young people were asked to think about any concepts that were missing from those identified in the academic literature. After the workshop, the draft definition was then finalized through discussion during a follow-up session involving the first reviewer and the young person co-facilitator.

## Results

**Fig. 1:**
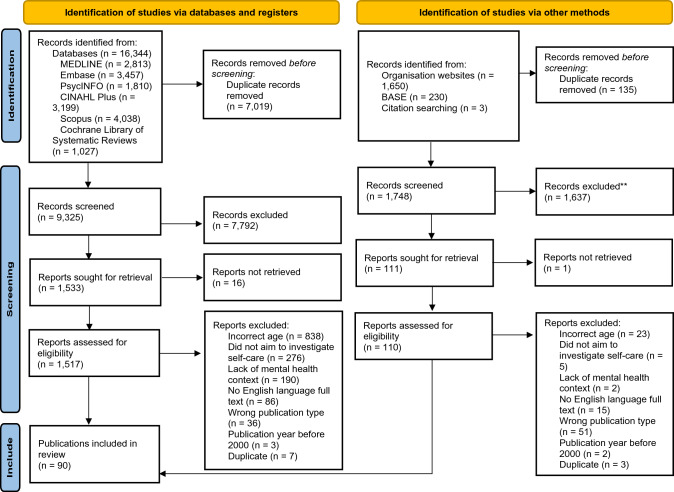
PRISMA diagram of study selection

The search process is detailed in Fig. 1. Searching across six academic databases identified 16,344 records, while gray literature searching identified 1883 records. After duplicates were removed, 11,073 records were screened using their titles and abstracts. After discussion of disagreements with the second screener, 1644 records were taken forward to the full text screening stage. Full texts were found for 1627 of these and 90 publications were considered to meet the inclusion criteria.

### Study characteristics

Of the 90 included publications, the large majority were primary research (*n* = 73), followed by discussion papers (*n* = 9), study registrations (*n* = 4), book chapters (*n* = 2) and secondary research (*n* = 2).

From population descriptions, publications most often investigated or discussed mental health self-care for university students (*n* = 57), followed by general child, adolescent or young people groups (*n* = 20), secondary school students (*n* = 10) and perinatal young women (*n* = 3). Among the 90 included publications an average participant age was given by 45, which for 6 studies fell between 11 and 14 years, for 11 studies between 15 and 18 years, for 14 studies between 19 and 22 years, and for 14 studies between 23 and 25 years. Study characteristics for each included publication are included in Table 1.

**Table 1:** Study characteristics table

References	Country	Publication type	Population	Age	Study aim
Akbarbegloo [30]	Iran	Primary research	Off-therapy childhood cancer survivors	Mean = 13.8 years (SD = 2.57)	To explore self-care needs of off-therapy childhood cancer survivors
Ashcraft [31]	USA	Primary research	Undergraduate nursing students	Mean = 23.5 years (SD = NR)	To understand self-care behaviors and perceptions following a self-care promotion intervention
Auttama [32]	Thailand	Primary research	University students	Median = 20 years (IQR = 1)	To investigate factors associated with self-esteem, resilience, mental health and psychological self-care
Ayala [33]	USA	Primary research	Medical students	Mean = 25.42 years (SD = 2.27)	To identify domains of health promotion and self-care attitudes of medical students
Aydin [34]	Turkey	Primary research	Mental health university students	Mean = 20.31 years (SD = 1.45)	To investigate the role of mindful self-care in predicting wellbeing
Baker [35]	USA	Primary research	Students of a human rights university program	Average = 23.4 years	To explore secondary trauma mitigation techniques, including self-care
Ball [36]	USA	Primary research	First year medical students	Average = 24.02 years (SD = 3.42)	To examine (1) health habits and (2) self-awareness and self-care interventions for health and emotional adjustment
Beaumont [37]	UK	Discussion paper	Undergraduate midwifery students	N/A	To investigate an intervention (which included self-care) to increase self-compassion and reduce self-criticism
Bender [38]	USA	Primary research	Undergraduate students	NR	To explore the relationships between attachment, resilience, self-efficacy and self-care
Betz [39]	USA	Primary research	Youth with spina bifida	Mean = 16.19 years (SD = 1.33)	To test a cognitive-behavioral program combined with spina bifida management on subjective wellbeing, role mastery and self-care
Brodsky [40]	USA	Primary research	First year medical students	NR	To evaluate an elective course involving the creation of a self-care plan
Brown [41]	USA	Discussion paper	Undergraduate medical students	N/A	To advocate for trauma-informed medical education, including trauma-informed self-care skills
Canty-Mitchell [42]	USA	Primary research	Inner-city adolescents	Mean = 15.92 years (SD = 1.29)	To explore the relationships between hope, life change events and self-care among inner-city adolescents
Chan [43]	Hong Kong	Primary research	Undergraduate counseling trainees	Range = 18–23 years	To investigate the impact of a mindfulness-based cognitive therapy program (which included self-care)
Clements [44]	USA	Primary research	Undergraduate social work students	Mean = 22.7 years (SD = NR)	To present a teaching approach with mutual aid-based stress-management groups, which included self-care skills
Crary [45]	USA	Primary research	Undergraduate nursing students	79% 20–24 years	To explore experiences of stress, coping, self-compassion, physical and emotional health and self-care
Cummins [46]	Australia	Primary research	First year undergraduate midwifery students	NR	To evaluate workshops designed to help student midwives discuss sensitive topics and develop self-care tools
Docherty-Skippen [47]	Canada	Primary research	Undergraduate nursing students	NR	To explore strategies used to teach self-care to nursing students
Drolet [48]	Canada	Discussion paper	Undergraduate social work students	N/A	To discuss teaching strategies to support wellness and self-care
Emery [49]	UK	Discussion paper	Undergraduate nursing students	N/A	To discuss coping strategies to nurture mental and physical wellbeing, including self-care strategies
Feng [50]	USA	Primary research	Young adult students	Mean = 23.5 years (SD = 4.8)	To explore the relationships between mindful self-care, perceived stress and quality of life
Fiodorova [51]	Canada	Primary research	Undergraduate psychology students	Mean = 18 years (SD = 1.08)	To investigate the impact of self-care on wellbeing
Florang [52]	USA	Discussion paper	Adolescents experiencing cyberbullying	N/A	To discuss self-care deficit theory as a framework to support adolescent victims of cyberbullying
Friedman [53]	USA	Discussion paper	Undergraduate students	N/A	To describe the implementation of a Mental Health Task Force, including undergraduate staff self-care training
Gordon [54]	Kosovo	Primary research	Secondary school students with PTSD symptoms	Mean = 16.3 years (SD = NR)	To investigate an intervention (including self-care) to improve symptoms of PTSD
Greene [55]	USA	Primary research	Undergraduate students	NR	To explore the impact of a mindfulness-based stress reduction course (including self-care)
Greeson [56]	USA	Primary research	Medical students	84% Years 1–3	To evaluate workshops covering self-care, mind–body medicine and mindfulness
Hassed [57]	Australia	Primary research	Undergraduate medical students	Mean = 18.77 years (SD = 1.1)	To test the impact of a Health Enhancement Program (including self-care strategies) on psychological distress and quality of life
Hensel [58]	USA	Primary research	Second year nursing students	NR	To explore the impact of an art therapy class to teach self-care and self-awareness
Hensel [59]	USA	Primary research	Undergraduate nursing students	Mean = 20.4 years (SD = 1.05)	To explore the relationship between self-care and stress during the development of a professional identity
Hofmeyer [60]	Australia	Primary research	Final year undergraduate nursing students	NR	To investigate the impact of a compassion module (including self-care) on compassion
Hosek [61]	USA	Clinical Trial registration	Youth newly diagnosed with HIV	Range = 16–24 years	To describe an HIV intervention (including self-care) to improve psychosocial adjustment of newly diagnosed youth
Ingram [22]	Canada	Primary research	Third year undergraduates	NR	To explore student perceptions of learning about mindfulness and self-care to improve wellbeing
Javed [62]	Pakistan	Primary research	Undergraduate nursing students	89.47% 16–25 years	To investigate the self-care habits of nursing students
Kao [63]	USA	Primary research	Pregnant women receiving public assistance	Mean = 22.4 years (SD = 4.4)	To investigate the impact of a psychoeducational intervention (including self-care)
Kearns [64]	UK	Primary research	Second year undergraduate teaching students	NR	To explore the impact of a course addressing attachment and how attachment related to student self-care
Kennison [65]	USA	Primary research	First year undergraduate students	Median = 19 years (IQR = NR)	To investigate the impact of an expressive writing intervention on self-care for stressful or traumatic experiences
Kirby [66]	UK	Primary research	Secondary school children	Mean = 12.4 years (SD = 0.7)	To test the impact of the Hopeful Minds program on hope, depression, anxiety, resilience, emotion regulation and coping (including self-care)
Kislyakov [67]	Russia	Primary research	University students	Mean = 20 years (SD = NR)	To investigate prosocial behavior strategies (including self-care) during COVID-19
Kongsuwan [68]	Thailand	Primary research	High school students showing moderate to high aggression	Mean = 13.44 years (SD = NR)	To evaluate the effectiveness of a violence prevention program involving self-care practices
Kramer [69]	USA	Discussion paper	Undergraduate nursing students	N/A	To describe a complementary and alternative medicine course as a means of self-care
Krasniqi [70]	USA and Canada	Primary research	Dental hygiene students	Mean = 25.3 years (SD = 6.6)	To explore self-care practices and their relationship with work hours and caregiver responsibilities
Lattie [71]	USA	Primary research	University students	Mean = 24.19 years (SD = 6.03)	To pilot a mental health app for university students (which included self-care)
Maddah [72]	Lebanon	Primary research	University students—low to medium socio-economic background	Mean = 21.01 (SD = 6.08; control) and 20.88 years (SD = 7.16; skills group)	To test the impact of a life skills-based intervention (including self-care) on BMI and mental health
Martin [73]	USA	Primary research	University seniors	NR	To investigate self-care practices to manage stress
Martorell-Poveda [20]	Spain	Primary research	Young people (depression diagnosis, distress and control groups)	Range = 17–21 years	To explore self-care strategies among young people to cope with distress
Mason [74]	USA	Primary research	First generation medical students	Mean = 25.64 years (SD = 3.29)	To compare levels of stress, self-care and quality of life between first and continuing generation medical students
McAllister [75]	Australia	Primary research	School nurses	N/A	To explore the need for and feasibility of a program for school nurses to support self-care among 13–14 year olds in order to prevent self-harm
McAllister [76]	Australia	Primary research	School mental health promotion program facilitators	N/A	To explore a training for school mental health promotion program facilitators which included supporting young people to develop self-care strategies
McAllister [77]	Australia	Discussion paper	Young people	12–13 years	To discuss the theory behind a program to improve young people’s self-care, sense of belonging, empathy and resilience
McGuinness [78]	Canada	Primary research	First year undergraduate students	Mean = 18 years (SD = 0.58)	To explore the impact of mindful self-care on flourishing
Meichenbaum [79]	USA	Chapter	LGBTQ Youth	N/A	To discuss resilience strategies among LGBTQ youth, including self-care
Moffett [80]	UK	Primary research	Undergraduate veterinary students	Mean = 19.8 years (SD = 2.8)	To explore the impact on resilience of a teaching intervention based on self-care
Moore [81]	Australia	Clinical Trial registration	Secondary school children	Range 11–14 years	To describe a martial arts training program (including self-care) for mental health
Moore [82]	USA	Primary research	Music therapy students	Mean = 23.44 years (SD = 6.66)	To investigate stress levels and self-care practices of music therapy students
Moore [83]	USA	Primary research	Undergraduate nursing students	NR	To explore the impact of a mental health nursing class (including self-care) presenting methods of stress reduction
Mosca [84]	USA	Primary research	Pre-health students	Mean = 24.4 years (SD = 4)	To explore self-care mechanisms and stress coping strategies for pre-health students
Moses [85]	Australia	Primary research	Undergraduate students	Mean = 20.87 years (SD = 3.31)	To investigate relationships between physical, cognitive and social self-care practices and psychological wellbeing
Muzik [86]	USA	Primary research	High-risk mothers	Mean = 23.7 years (SD = 5.3)	To explore the feasibility and acceptability of an intervention (including self-care) for high-risk mothers
Muzik [87]	USA	Primary research	At-risk mothers of young children	Mean = 23.71 years (SD = 6.14)	To explore treatment engagement during an intervention (including self-care) for at-risk mothers
Nafiseh [24]	Iran	Primary research	Adolescents	Range = 14–20 years	To describe spiritual self-care among Iranian adolescents
Nash [88]	USA	Primary research	Middle school students	Mean = 12.8 years (SD = NR)	To investigate the impact of a peer education and support group program on self-care resources
Newcomb [23]	Australia	Primary research	Undergraduate social work and human services students	Median = 23.5 years (IQR = NR)	To understand how students with adverse childhood experiences learn about self-care
Ng [89]	Hong Kong	Primary research	Final year university students	Range = 18–25 years	To explore a body-mind-spirit intervention (including self-care) which aimed to promote holistic wellbeing
Ng [90]	Hong Kong	Primary research	First year university students	Mean = 21.4 (SD = 2; control) and 22.6 years (SD = 3.3; BMS group)	To test the impact of a body–mind–spirit intervention (including self-care) on holistic wellbeing
O'Brien [91]	USA	Primary research	Sexual minority adolescents	Mean = 17.53 years (SD = 1.02)	To understand self-care practices among sexual minority adolescents during COVID-19
Otsuka-Ono [92]	Japan	Primary research	Female high school students	Mean = 16.5 years (SD = NR)	To identify self-care strategies for premenstrual distress
Pakenham [93]	Australia	Primary research	Third year psychology students	Mean = 23.15 years (SD = 6.76)	To evaluate a personal practice-informed psychotherapy curriculum, including impacts on self-care
Pryjmachuk [94]	UK	Secondary research	Children and young people	63% included studies 11–25 years	To investigate empirical studies on mental health self-care support
Rababah [95]	Jordan	Primary research	Undergraduate nursing students	Average = 21.5 years (SD = 2.24)	To examine the relationships between mindfulness and health promotion
Redwood [96]	USA	Primary research	First year medical students	NR	To evaluate a stress-management program (including self-care) for first year medical students
Risdon [97]	Canada	Chapter	Early year medical students	N/A	To describe a curriculum (including self-care) aiming to improve resilience
Roy [98]	UK	Primary research	First year medical and nursing students	NR	To evaluate the delivery of a wellbeing workshop, including self-care
Rusch [99]	USA	Primary research	School mental health staff	N/A	To explore school student mental health care needs, including self-care
Sarkhani [100]	Iran	Clinical Trial registration	Adolescent girls	Range = 12–15 years	To describe a self-care intervention aiming to support health promotion
Schmidt [101]	UK	Primary research	Adolescents with bulimia nervosa or eating disorder not otherwise specified	Mean = 17.9 (SD = 1.6; family therapy) and 17.4 years (SD = 1.8; guided self-care)	To investigate the impact of family therapy and CBT guided self-care among adolescents with bulimia nervosa or eating disorder not otherwise specified
Schmidt [102]	UK	Primary research	Adolescents with bulimia nervosa or eating disorder not otherwise specified (EDNOS)	Mean = 17.7 (SD = 1.7; bulimia nervosa) and 17.4 years (SD = 1.7; EDNOS)	To explore whether adolescents with bulimia nervosa differed from those with EDNOS on eating disorder or outcome characteristics (between family therapy and CBT guided self-care)
Sharif Nia [103]	Iran	Primary research	Medical and related subjects students	Mean = 21.02 years (SD = 2.014)	To explore the relationship between religious coping and self-care behaviors
Slemon [19]	Canada	Secondary research	Nursing students	85% undergraduate student studies	To investigate how the concept of self-care has been used in the nursing education literature
Slonim [104]	Australia	Primary research	Medical students	Mean = 21.82 years (SD = 3.62)	To examine relationships between self-care behaviors, mindfulness and distress
Snyder [105]	USA	Primary research	Undergraduate nursing students	NR	To explore the impact of teaching positive coping skills and self-care strategies
Stanfield [106]	USA	Primary research	Camp counselors	Average = 20.33 years (SD = 2.35)	To examine the impact of self-care activities, self-compassion, stress, values activities and burnout on compassion satisfaction
Thomas [107]	USA	Primary research	First year medical students	NR	To evaluate a pilot self-care program for first year medical students
Town [108]	UK	Scoping review protocol	Adolescents	N/A	To investigate the concepts of self-management, self-care and self-help for adolescents with emotional problems
Tuong [109]	Vietnam	Primary research	Adolescents	Mean = 14.63 years (SD = 1.08)	To explore care-competencies including receiving care, self-care and extending care and their relationship with wellbeing
Ul Huda [110]	Pakistan	Primary research	Adolescent boys	NR	To examine adolescent boys’ perceptions and practices of self-care during puberty
Valizadeh [111]	Iran	Primary research	Adolescent cancer survivors	Mean = 15 years (SD = 2.51)	To explore perceptions of self-care needs among adolescent cancer survivors
Yanaz [112]	Turkey	Primary research	Children with cystic fibrosis and healthy peers	Mean = 11.53 (SD = 2.86; cystic fibrosis) and 11.76 years (SD = 3.15; control)	To evaluate the impact of COVID-19 on family environment, self-care practices, peer relations, psychological health and coping
Zarimoghadam [113]	Iran	Primary research	Students referred to school counseling	NR	To investigate the impact of mental health self-care education on mental health and academic motivation
Zurbriggen [114]	USA	Discussion paper	Undergraduate university students	N/A	To discuss secondary and vicarious traumatization when teaching courses about trauma, including strategies to mitigate these risks (e.g., self-care)

UK, United Kingdom; USA, United States of America; NR, Not Reported; N/A, Not Applicable

Among the included publications, 38% defined self-care (*n* = 34) and 53% measured self-care (*n* = 48). Furthermore, 30% of included studies both defined and measured self-care (*n* = 27) while 39% neither defined or measured (*n* = 35).

### Part 1: Conceptualizations of self-care in the academic literature

To understand academic conceptualizations of self-care in the young people’s mental health and wellbeing literature, referenced definitions of self-care are presented first, followed by content analysis of self-care definitions and measures used by the included publications. Of the 34 publications which defined self-care, they cited a total of 51 other definitions. Only seven of these were cited by more than one publication and are presented in Table 2.

**Table 2:** Definitions of self-care referenced more than once

Reference cited	*k*	Definition
Orem [13, 115–119]	5	“The practice of activities that individuals initiate and perform on their own behalf in maintaining life, health and well-being” [13]
World Health Organization [14, 120, 121]	4	“Self-care is the ability of individuals, families and communities to promote health, prevent disease, and maintain health and to cope with illness and disability with or without the support of a health-care provider” [14]
Cook-Cottone et al. [122–124]	3	“Mindful self-care is seen as the active practice of behaviors that facilitate and maintain attunement and balance among the internal aspects of self and the external aspects of self” [122]
Pender et al. [125]	2	“Individual responsibility to promote one’s health and well-being” [125]
Myers et al. [126]	2	“Self-care practice may be defined as engagement in behaviors that maintain and promote physical and emotional wellbeing and may include factors such as sleep, exercise, use of social support, emotion regulation strategies, and mindfulness practice” [126]
Salloum et al. [127, 128]	2	“Self-care is often defined and measured by different activities and domains that promote wellbeing. Domains may include physical, emotional, psychological, leisure, spiritual (i.e., personal self-care strategies), and professional activities” [127]
Newell and Nelson-Gardell [129]	2	“Professional self-care can be defined as the utilization of skills and strategies by social workers to maintain their own personal, familial, emotional, and spiritual needs while attending to the needs and demands of their clients” [129]

Considering the definitions as presented in the text of the 34 defining publications, content analysis identified the most frequently used concepts as health and wellness promotion (*n* = 22, 65%), maintaining health or wellbeing (*n* = 18, 53%), illness prevention (*n* = 7, 21%), managing illness (*n* = 7, 21%), and self-awareness (*n* = 7, 21%). The least frequently mentioned concepts in these definitions were engaging in a caring relationship (*n* = 1, 3%), improving quality of life (*n* = 1, 3%), enhancing resilience (*n* = 1, 3%) and improving life satisfaction (*n* = 1, 3%).

Looking at specific activities mentioned in the self-care definitions, the most common included exercise (*n* = 7, 21%), supportive relationships (*n* = 7, 21%), personal care (*n* = 5, 15%) and spiritual or religious activities (*n* = 5, 15%), with creativity (*n* = 1, 3%), help-seeking (*n* = 1, 3%), positive psychology techniques (*n* = 1, 3%) and psychoeducation (*n* = 1, 3%) mentioned the least often.

### Measurement of self-care

Of the included papers 48 measured self-care in some way, with the most common method being validated measures (*n* = 20, 42%) followed by project-specific questionnaires (*n* = 10, 21%), interview questions (*n* = 8, 17%), non-validated measures (*n* = 4, 8%), systematic review search terms (*n* = 3, 6%), instructions for a written activity (*n* = 2, 4%) and a deductive coding framework (*n* = 1, 2%).

Among the 34 publications which used validated measures, project-specific questionnaires or non-validated measures, a total of 19 concepts were measured to investigate self-care. The most common overarching concepts were a direct measurement of self-care (*n* = 16, 47%) and health promotion (*n* = 5, 15%), with 12 concepts each used by only one included paper.

Ten publications measured self-care either through interview questions or instructions for a written activity. Most of these asked generally about self-care (*n* = 6, 60%), while 40% (*n* = 4) asked about managing stress or emotional distress, 30% (*n* = 3) asked about daily time use and spiritual or religious practices, 20% (*n* = 2) about exercise, health and wellness promotion, mindfulness and personal care, and 10% (*n* = 1) about improving quality of life, maintaining wellbeing, general needs, and supportive relationships.

The two publications which captured self-care through systematic review search terms took different approaches, with one solely searching for the term self-care and the other employing a wide range of terms including ‘self management’, ‘self help’, ‘self report’, ‘self monitor’, self medicate’, ‘self administer’, ‘self treat’, and ‘self control’. Finally, the one publication which assessed self-care through a deductive coding framework used a framework based on mindfulness, physical exercise, food habits, social support and sleep hygiene as aspects of self-care.

### Conceptualization of self-care across definitions and measures

Considering the concepts used across both definitions and measures of self-care, the most frequent were health and wellness promotion (*n* = 25, 45%), self-care or self-care agency (*n* = 24, 44%), maintaining health or wellbeing (*n* = 19, 35%) and personal care/healthy lifestyle activities (*n* = 16, 29%). The self-care or self-care agency concept is applicable only to the measures of self-care, where the overall concept measured was described as self-care or self-care agency, or where other types of measurement specifically asked about the term ‘self-care’. The least frequently mentioned concepts looking across both definition and measurement domains were help-seeking (*n* = 1, 2%), engaging in a caring relationship (*n* = 1, 2%), improving life satisfaction (*n* = 1, 2%), psychoeducation/positive psychology techniques (*n* = 1, 2%), life skills (*n* = 1, 2%), self-management (*n* = 1, 2%) and sleep hygiene (*n* = 1, 2%). These concepts identified by the systematic review were subsequently displayed as a set of 42 tiles in the online Patient and Public Involvement workshop.

### Part 2: Defining self-care with young people

#### Patient and Public Involvement workshop

The Patient and Public Involvement workshop aimed to evaluate the findings of the systematic review and extend these findings by working with young people to co-develop a definition which they felt to be relevant to their lived experience of mental health self-care. The workshop firstly elicited initial thoughts on the meaning of self-care, which included a number of broader concepts including ‘listening to my body’, ‘benefit mental health’, ‘unique’, ‘personal’, ‘paced’, ‘self-management’ and ‘strategies’. Many examples of self-care activities were also shared, such as ‘eating real food’, ‘cleaning’, ‘hobbies’, ‘re-energizing activities’, ‘sleep’ and ‘exercise’, as well as some outcomes of self-care, such as ‘relax’, ‘treating yourself’, ‘no mental strain’, and ‘enjoyment’. From a set of voting options, 4 of 5 workshop attendees indicated that they felt that self-care was best described as a ‘continuous process’, with one participant indicating that self-care was ‘a set of independent activities’ and none selecting ‘something else’.

The concepts and activities described by either the definitions or measures of self-care identified by the systematic literature review were presented as a set of 42 tiles in an online rating activity. During the activity, the workshop attendees indicated a set of eight definition concepts as ‘Completely relevant’ and eleven concepts as ‘Slightly relevant’, while eight concepts were uncoded at the end of the set time (positive psychology, supportive relationships, mindfulness, daily routine, time outdoors, spiritual activities such as yoga and meditation, creative activities and improving quality of life). Categorizations of the definition concepts are presented in Table 3. Two concepts (self-management and holistic wellbeing) were given an ‘other’ code, which the young people explained as indicating that these concepts were seen as synonymous with self-care rather than concepts able to define self-care.

**Table 3:** Categorizations of definition elements by PPI workshop attendees

Completely irrelevant	Slightly irrelevant	Slightly relevant	Completely relevant
Empowerment	Health promotion	Psychoeducation	Self-compassion
Recovery	Help-seeking	Sleep hygiene	Monitoring wellbeing
Coping	Purpose	Supportive structures	Self-awareness
Self-improvement	Caring relationships	Managing illness	Balance
Life skills	Maintaining wellbeing	Maintaining health	Improving life satisfaction
Religious activities	Monitoring health	Relaxation	Personal care (e.g., personal hygiene)
Preventing illness		Resilience	Meeting mental health needs
		Wellness promotion	Entertainment activities (e.g., reading, watching TV)
		Managing stress	
		Healthy lifestyle activities (e.g., exercise, healthy eating)	
		Meeting general needs	

During discussion, the young people highlighted self-compassion and monitoring as key aspects. Balance was identified as an overall aim of self-care, as finding balance was seen as a tangible everyday process that young people engaged in through self-care, to varying degrees of success. Although some activities were indicated as ‘Completely’ or ‘Slightly’ relevant, the young people felt that these should not be included in the definition as specific self-care activities, such as particular personal care or entertainment activities, will be unique to each person’s experience of self-care.

A meeting with the young person co-facilitator was then used to develop the definition into a final draft. This final draft defines self-care as:

A continuous, individual process that uses specific strategies guided by self-awareness to meet mental health needs. Self-care will be unique to each person, involving a self-compassionate approach to find emotional balance and develop positive strategies to promote mental health and wellbeing.

## Discussion

This study aimed to identify current conceptualizations of self-care in the young people’s mental health and wellbeing literature and to facilitate young people to respond to these findings through a Patient and Public Involvement workshop. The systematic review findings indicate that although this is an active and growing field, there does not seem to be a consistent foundation of conceptual understanding. A definition of mental health self-care co-developed with young people emphasizes the importance of self-awareness and self-compassion in developing specific strategies to achieve emotional balance and support mental health and wellbeing outcomes.

### Conceptualization of young people’s mental health self-care in the academic literature

This systematic review identified a wide range of concepts which have been employed when defining and measuring mental health self-care. The underpinning concepts used to define self-care for this age group were largely inconsistent, ranging from health promotion and improving quality of life to purpose and self-improvement, with 18 different concepts described among this sub-sample of papers. These findings are generally consistent with previous reviews, which have commented on a lack of consensus on the definition and scope of self-care when looking at research focused on older adults [17] and that over time, definitions of self-care have generally become broader and less specific [16]. Overall, these findings suggest that although the included papers all discuss or investigate mental health self-care for young people, it is likely that fundamental differences exist in relation to the specific concepts under discussion or investigation.

Perhaps reflecting that these definitions are not a perfect fit for publications discussing self-care for young people’s mental health, no single definition was cited by more than five of the 34 publications which defined self-care. Despite this, 22 of the 34 definitions mentioned health and wellness promotion and health promotion was the second most common concept investigated by self-care measures and questionnaires. This is consistent with a review of self-care definitions for long-term health conditions, where the term was found to include health promotion, prevention of disease and accidents, limitation of illness and restoration of health [130]. In this review, health promotion was most often used as a measurement of self-care through the Health Promoting Lifestyle II [131]. The subscales of this measure include spiritual growth, health responsibility, interpersonal relations, nutrition, physical activity and stress management [132], with health promoting behavior defined as “an expression of the human actualizing tendency, is directed toward sustaining or increasing the individual’s level of wellbeing, self-actualization, and personal fulfillment” [131]. Considering these subscales and the definition of health promotion, the current conceptualizations of self-care have yet to establish independence from health promotion and how self-care is specifically applicable in the context of mental health.

The suggestion that a single definition of self-care many not be sufficient for all purposes [18] is also reflected in the varying population groups of included papers, which influence the extent to which the identified conceptualizations are applicable to broader groups of young people. The majority of included studies in this review focused on university student groups, with a large proportion addressing self-care for students entering demanding healthcare or helping professions. As indicated by a scoping review focused on the meaning of self-care for nursing students, self-care for these groups can be more concerned with managing work-related stress to enable optimal professional practice [19], rather than living with mental health difficulties. Therefore, distinguishing self-care which is used to address mental health difficulties from self-care for general wellbeing may be helpful to enable the development of specific understandings of self-care for these differing purposes.

Studies on university student groups were included in this review through the older portion of the age range. This could mean that some findings reflect self-care at a specific life stage in the context of greater independence. The involvement of students from healthcare programs also brings a specific context, where these students are perhaps more aware of healthcare management and the studies typically investigated more directed forms of self-care during a degree course. Again, this may bring a specific understanding of self-care which further research will need to explore in relation to its applicability to self-care as a more self-directed process. The studies included in this review explore self-care in both contexts and while it does not appear that separate but coherent conceptualizations currently exist, potential differences between the two is a valuable area of future research. It may be the case that future development of the self-care support delivered as part of university programs would benefit from a deeper understanding of the self-care meanings and needs of the young people involved.

A clearer focus on mental health and wellbeing does appear to be present in the interview questions and instructions for a written activity. Although only ten publications measured self-care in this way, questions most often included asking about self-care generally, followed by how participants managed stress or emotional distress, how they spent their daily time and about spiritual or religious practices. Questions about how participants spend their daily time may not directly relate to mental health or wellbeing, but do seem to share some conceptual similarity with Cook-Cottone’s [15] definition of self-care as a daily process of caring for physiological and emotional needs including making alterations to daily routines. Additionally, a review of self-care in mental health services has highlighted knowledge, self-efficacy and capacity as key determinants of the self-care strategies that are available to each person [12]. This review recognizes that engaging in self-care requires the availability of a range of resources, including time, capacity to engage in self-care activities and access to the activities themselves. This was also partially acknowledged in the PPI workshop, where self-care was felt to be unique to each person and will likely reflect both the young person’s resources and access to support. A combination of self-care concepts focused on mental health, how these concepts are enacted in daily life and the factors influencing which self-care activities a young person is able to use are all necessary in order to provide a more complete understanding of young people’s mental health self-care.

Overall, it seems that a definition of self-care for mental health and wellbeing is emerging, but that substantial conceptual inconsistency still exists. This has resulted in difficulties in distinguishing self-care from other concepts and relating self-care activities to an organizing conceptual foundation. Developing an understanding of self-care specifically for young people’s mental health and wellbeing rather than using a very broad definition covering the spectrum of physical health, wellbeing and mental health appears necessary, especially when considering how to support young people with self-care.

### Co-producing a definition of mental health self-care with young people

The Patient and Public Involvement workshop enabled the elements of self-care conceptualizations identified through the systematic review to be evaluated against lived experience, with the most relevant concepts organized into a definition of self-care. Involvement of young people at this stage was essential given that there is no ‘ideal’ standard of self-care against which to evaluate the identified concepts. As well as integrating both academic and lived experience viewpoints, this definition also seeks to bring greater clarity to ‘how’ self-care takes place in everyday life, something often missing from existing definitions.

The workshop indicated that academic conceptualizations of mental health self-care may not accurately reflect young people’s understandings and experiences. Of the most commonly identified concepts from the academic literature (health and wellness promotion, maintaining health and wellbeing, personal care and healthy lifestyle activities), only personal care was rated as ‘Completely relevant’ by the workshop attendees. The role of personal care and physical health has been raised in previous research asking young people about how they look after their mental health, for example with interviews with LGBTQ + young people identifying strategies including hygiene, exercise and drinking enough water [21]. However, the young people attending the workshop did not feel that specific activities such as these should be included in the definition, as they were unlikely to be relevant to all young people engaged in mental health self-care. Indeed, this is consistent with the findings from LGBTQ + young people, where only one activity was mentioned by more than 50% of participants and personal hygiene activities were mentioned by 10% [21]. Other concepts rated as ‘Completely relevant’ in the PPI workshop did not necessarily reflect those most often mentioned in academic definitions, such as improving life satisfaction which was raised in only one included study.

Additionally, during open discussion, the workshop attendees highlighted missing elements, such as the unique nature of mental health self-care to each person, which forms a key part of the presented definition and helps to clarify how the overall definition relates to specific individuals. Viewing self-care as unique to each person allows for differences depending on individual needs, values and aims for mental health self-care. While the young people involved in the workshop felt that focusing on daily outcomes of finding balance as a step toward positive mental health most relevant, this could be enacted in a range of ways. This allows the process suggested by the co-produced definition to be shaped by young people’s individual understandings of their mental health, rather than acting as a prescriptive set of activities.

In comparison to previous research which has asked young people about their understandings of self-care for mental health or wellbeing [22–24], this definition similarly highlights self-awareness as a key concept, but does not include stress management. This may reflect the different challenges faced between students entering healthcare professions and the workshop attendees who had experience of mental health difficulties, where stress was seen as ‘slightly’ but not ‘completely relevant’ to a definition of self-care. Stress management was a relatively common element used across self-care conceptualizations identified by the systematic review, perhaps due to the high number of university-based studies and also to the inclusion of stress management as part of the Health Promoting Lifestyle Profile II [131], which has been relatively frequently used as a measure of self-care. Additionally, these contrasting conceptualizations of self-care serve to underline the findings of Newcomb, et al. [23], that the meaning of self-care is not self-explanatory and indeed may need careful definition for the specific context.

The context of this co-developed definition will help future research on young people’s mental health self-care to start from a conceptual basis that is relevant to young people’s experiences of self-care. As discussed, self-care may be understood differently in different contexts, so the populations where this definition is relevant are likely to be limited to young people with experience of mental health difficulties. While the systematic review included global research, the review was limited to English language publications and the PPI workshop was carried out in the UK, meaning that the co-produced definition is also specific to a UK context. This definition is intended as a starting point and further adaptation based on lived experience should be considered, including for young people not in contact with services. Further research should explore how this definition is put into practice by young people and how this type of self-care is related to mental health and wellbeing, which could enable the development of better mental health and wellbeing support for young people, based in specific self-care needs and understandings.

There are limitations to consider for the systematic review, first that the review aimed to identify publications focusing on young people up to the age of 25, but may have missed publications which only described their sample as adults. Second, the conceptual confusion which prompted this review has also been apparent in the lack of clarity among many publications in the purpose of self-care, which presented challenges during the screening process and in interpreting the various ways in which self-care has been conceptualized. This review also has some important strengths, including the relatively wide age range of 11–25 years which allowed for an understanding of the depth of evidence at various ages. The predominant focus on university students among the included papers indicates a lack of current research focus on mental health self-care for children and young people and therefore potentially limited applicability of the concepts to these age groups.

Considering the PPI workshop, facilitation of the session by the researcher, young people’s participation specialist and young person co-facilitator was greatly beneficial. The Lundy Model of Child Participation [29] emphasizes the need for appropriate conditions and support for young people to express their views, which this combination of co-facilitators seemed to enable. Another key aspect of the Lundy model involves providing an inclusive space for all young people to give their views on issues that affect them. The support provided by the mental health charity these young people were already engaged with helped to enable these young people to access the session and share their views on mental health research, though it should also be recognized that this did limit participation to young people already interested and engaged with mental health research and services.

## Conclusion

Conceptualizations of self-care for young people’s mental health or wellbeing in the academic literature are currently inconsistent and often lacking entirely. This presents a challenge to building an evidence base around self-care support for young people and hinders the integration and applicability of current research.

The present research represents an initial step to address these issues. The co-developed definition draws from concepts identified in the academic literature, but also enabled young people with experience of mental health difficulties to evaluate these concepts and to add concepts they felt to be important. The definition presented here is intended as a starting point to conduct research in this area which is relevant and applicable to young people’s understanding and practice of mental health self-care.

Whether or not a young person chooses to or is able to access professional mental health care, self-care is accessible to all young people. The development of evidence-based information and guidance about self-care processes has the potential to support young people to develop self-awareness skills and strategies to help maintain or restore emotional balance, potentially enabling a greater ability to manage day-to-day life while experiencing mental health difficulties.

## Supplementary Information

Below is the link to the electronic supplementary material.

Supplementary file 1 (DOCX 23 KB)

## Data Availability

Not applicable.
