# Supplementary material for: Defining young people’s mental health self-care: a systematic review and co-development approach
Source: Eur Child Adolesc Psychiatry. 2023 Nov 10;33(11):3765–85. doi: 10.1007/s00787-023-02320-7 (PMC11588886; doi:10.1007/s00787-023-02320-7)
Supplement: Supplementary file 1 — (DOCX 23 KB) [file 787_2023_2320_MOESM1_ESM.docx]

## Article title: Defining young people’s mental health self-care: A systematic review and co-development approach

**Journal name**: European Child & Adolescent Psychiatry

## Author names

Alex Truscott

Daniel Hayes

Tom Bardsley

Disha Choksi

Julian Edbrooke-Childs

## Corresponding author affiliation and email address

Alex Truscott

Evidence Based Practice Unit, University College London and Anna Freud National Centre for Children and Families. 4-8 Rodney St, London N1 9JH

a.truscott@ucl.ac.uk

## Online Resource 1

### MEDLINE Search Strategy

| 1 | Self Care/ |
| --- | --- |
| 2 | self-care.ti,ab,kf |
| 3 | (self adj4 care).ti,ab,kf |
| 4 | (self adj4 caring).ti,ab,kf |
| 5 | self-caring.ti,ab,kf |
| 6 | 1 or 2 or 3 or 4 or 5 |
| 7 | Adolescent/ |
| 8 | Adolescent Development/ |
| 9 | Adolescent Behaivour/px [Psychology] |
| 10 | Adolescent Health/ |
| 11 | Adolescent Psychiatry/ |
| 12 | Psychology, Adolescent/ |
| 13 | Child/gd, px, th [Growth & Development, Psychology, Therapy] |
| 14 | child behavior/ or problem behavior/ |
| 15 | Child Development/ |
| 16 | Child Psychiatry/ |
| 17 | Psychology, Child/ |
| 18 | Young Adult/px [Psychology] |
| 19 | exp Students/px [Psychology] |
| 20 | Pediatrics/px, th [Psychology, Therapy] |
| 21 | "young person".mp |
| 22 | "young people".mp |
| 23 | "young adult*".mp |
| 24 | "emerging adult*".mp |
| 25 | adolescen*.mp |
| 26 | child*.mp |
| 27 | student*.mp |
| 28 | teen*.mp |
| 29 | youth*.mp |
| 30 | p?ediatric.mp |
| 31 | juvenile*.mp |
| 32 | kids.mp |
| 33 | minors.mp |
| 34 | undergraduate*.mp |
| 35 | pupil*.mp |
| 36 | 7 or 8 or 9 or 10 or 11 or 12 or 13 or 14 or 15 or 16 or 17 or 18 or 19 or 20 or 21 or 22 or 23 or 24 or 25 or 26 or 27 or 28 or 29 or 30 or 31 or 32 or 33 or 34 or 35 |
| 37 | Mental Health/ |
| 38 | mental disorders/ or exp anxiety disorders/ or exp "bipolar and related disorders"/ or exp "disruptive, impulse control, and conduct disorders"/ or exp dissociative disorders/ or exp "feeding and eating disorders"/ or exp mood disorders/ or exp neurotic disorders/ or exp personality disorders/ or exp "schizophrenia spectrum and other psychotic disorders"/ or exp substance-related disorders/ or exp "trauma and stressor related disorders"/ |
| 39 | Psychopathology/ |
| 40 | Anxiety/pc, px, th [Prevention & Control, Psychology, Therapy] |
| 41 | Emotional Regulation/ |
| 42 | Psychological Distress/ |
| 43 | "mental health*".mp |
| 44 | "mental illness*".mp |
| 45 | (mental adj3 disorder*).mp |
| 46 | (mental adj3 condition*).mp |
| 47 | (mental adj3 problem*).mp |
| 48 | psychiatr*.mp |
| 49 | ("psychological distress" or "psychological problem*" or "psychological wellness" or "psychological wellbeing" or "psychological trauma" or "psychological symptom*" or "psychological stress*" or "psychological quality of life").mp. |
| 50 | psychopathology.mp |
| 51 | ("psychosocial health" or "psychosocial functioning" or "psychosocial distress" or "psychosocial stress*" or "psychosocial difficult*").mp. |
| 52 | internali?ing.mp |
| 53 | "emotional problem*".mp |
| 54 | "emotional disorder*".mp |
| 55 | "mood disorder*".mp |
| 56 | "affective disorder*".mp |
| 57 | anxiety.mp |
| 58 | stress* |
| 59 | depress*.mp |
| 60 | OCD.mp |
| 61 | "obsessive compulsive".mp |
| 62 | PTSD.mp |
| 63 | posttraumatic.mp |
| 64 | post-traumatic.mp |
| 65 | "traumatic stress".mp |
| 66 | phobi*.mp |
| 67 | "panic disorder*".mp |
| 68 | dissociat*.mp |
| 69 | "eating disorder*".mp |
| 70 | anorexi*.mp |
| 71 | bulimi*.mp |
| 72 | "binge eating disorder" |
| 73 | bipolar.mp |
| 74 | psychosis.mp |
| 75 | schizophren*.mp |
| 76 | "personality disorder*".mp |
| 77 | externali?ing.mp |
| 78 | "conduct disorder*" |
| 79 | "behavio?r disorder*".mp |
| 80 | "oppositional defiant disorder".mp |
| 81 | (substance adj3 disorder*).mp |
| 82 | "substance abuse".mp |
| 83 | (alcohol adj3 disorder*).mp |
| 84 | wellbeing.mp |
| 85 | well-being.mp |
| 86 | 37 or 38 or 39 or 40 or 41 or 42 or 43 or 44 or 45 or 46 or 47 or 48 or 49 or 50 or 51 or 52 or 53 or 54 or 55 or 56 or 57 or 58 or 59 or 60 or 61 or 62 or 63 or 64 or 65 or 66 or 67 or 68 or 69 or 70 or 71 or 72 or 73 or 74 or 75 or 76 or 77 or 78 or 79 or 80 or 81 or 82 or 83 or 84 or 85 |
| 87 | 6 and 36 and 86 |
| 88 | limit 86 to yr="2000 -Current" |
